# Terahertz virus-sized gold nanogap sensor

**DOI:** 10.1515/nanoph-2022-0706

**Published:** 2023-01-03

**Authors:** Gangseon Ji, Hwan Sik Kim, Seong Ho Cha, Hyoung-Taek Lee, Hye Ju Kim, Sang Woon Lee, Kwang Jun Ahn, Kyoung-Ho Kim, Yeong Hwan Ahn, Hyeong-Ryeol Park

**Affiliations:** Department of Physics, Ulsan National Institute of Science and Technology (UNIST), Ulsan 449419, Republic of Korea; Department of Physics, Department of Energy Systems Research, Ajou University, Suwon 16499, Republic of Korea; Department of Physics, Research Institute for Nanoscale Science and Technology, Chungbuk National University, Cheongju 28644, Republic of Korea

**Keywords:** atomic layer lithography, nanogap, refractive index sensing, terahertz sensing, terahertz time-domain spectroscopy, virus

## Abstract

We demonstrated an ultra-sensitive terahertz virus detection method combined with virus-sized gold nanogaps filled with Al_2_O_3_. Large-area high-density 20 nm-gap rectangular loop structures, containing a resonant frequency in the terahertz range, were fabricated on a 4-inch wafer using atomic layer lithography. When target viruses with a 60 nm diameter were located on the nanogaps, we observed a significant redshift of the resonant peak already with an average number of about 100 viruses per unit loop due to the strong field confinement and enhancement near the gap. Furthermore, when the virus was tightly attached to an etched gap like a bridge connecting metals, its sensitivity is doubled compared to the unetched gap, which resulted in 400% more resonance frequency shift per single virus particle than our previous work. Full-wave simulations and theoretical calculations based on modal expansions were in good agreement with the experiments, revealing that the resonant transmission spectrum was mostly determined by the change in refractive index in a two-dimensional-like optical hotspot near the nanogap. A further step could be taken to increase sensitivity by tuning nanogap-loops to the absorption frequencies associated with the intermolecular vibrational modes of the viruses and fingerprinting them as well.

## 1 Introduction

Detecting and characterizing biomolecules such as virus, protein, and DNA has become a vital part of the response to the recent COVID-19 pandemic. Various testing methods such as the polymerase chain reaction (PCR) and several alternative antigen and antibody tests have been further developed and improved in recent years [1], [2], [3], [4]. The PCR tests are considered as the gold standard because the high-sensitivity PCR tests are almost 100% accurate in spotting infected people, however, such tests take hours to provide the results [3]. Antigen tests give results in less than 30 min, but the tests require tens of thousands of virus particles per microliter to produce a positive result [5]. For another alternative diagnostic method, various light-based detection methods have been developed in a broadband wavelength range from X-ray to microwave [6], [7], [8], [9], [10], [11], [12]. Especially, terahertz sensing of chemical and biological molecules has attracted strong attention in optical sensing communities due to their advantages of fast, non-destructive, label-free, and ultra-small quantity detection [7, 8, 13], [14], [15], [16], [17]. However, due to the size mismatch between target viruses with a diameter below 100 nm [18], [19], [20] and the terahertz wavelength of a few hundreds of microns, it still remains a challenge to detect a tiny amount of viruses.

To overcome the size mismatch issue, terahertz metamaterials [21], [22], [23], [24], such as split-ring resonators [7, 14, 25], slot antenna [8, 26, 27], and graphene metasurface [28], have been suggested for sensing the target viruses. These subwavelength structures enable perception of the dielectric constant change within localized electric field areas and allow extreme light–matter interaction. However, it might be difficult to reduce the size of the gap to a few nanometers and simultaneously pattern over a large area, using standard lithography techniques, such as electron-beam lithography and photolithography. Recently, atomic layer lithography that can resolve the aforementioned challenges was developed for the fabrication of nanogaps with a width below 1 nm and packing 150,000 such devices on a 4-inch wafer [29], [30], [31]. Furthermore, it allows decreasing the size of the gap down to 1 nm (∼*λ*/10^6^) leading to extreme field confinement and enhancement near the gap in the terahertz frequency regime [29] and detecting the change of refractive index caused by 1 nm-thick dielectric overlayers [32].

**Figure 1:**
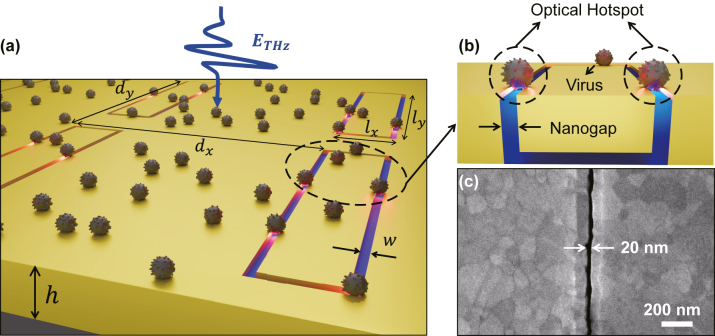
Terahertz virus-sized gold nanogap. (a) Schematic of the gold nanogap-loop array (*l*_*x*_ = 10 μm, *l*_*y*_ = 40 μm, *d*_*x*_ = 100 μm, *d*_*y*_ = 50 μm, and *h* = 100 nm) coated with PRD1 viruses. The electric field polarization of the terahertz wave is perpendicular to the long axis of each loop. (b) Enlarged view of the PRD1 virus particle onto the etched gap with a width (*w*) of 20 nm. Strong near-field confinement in the vicinity of nanogaps called an optical hotspot facilitates the sensitive detection of gap-sized PRD1 viruses. (c) Top-view scanning electron microscope (SEM) image of a 20 nm gold nanogap.

Here we demonstrated terahertz sensing of a tiny amount of PRD 1 which is double-stranded DNA bacteriophage and prototype of the Tectivirus with 10 species in five genera [33]. We utilized the high-density gold nanogaps with a gap width of 20 nm, which is about three times smaller than the size (∼60 nm) of the target virus, as depicted in Figure 1(a). In the vicinity of optical hotspots near the nanogaps, strong light–matter interaction is promised, and the nanogaps were uniformly extended to a hundred micrometers-long loop (*l*_*x*_ = 10 μm and *l*_*y*_ = 40 μm) to sustain strong terahertz resonances (Figure 1(a) and (b)). As shown in Figure 1(c), our nanogap structure has a slanted wall on the top of gap region, virus particles can tightly fit as a bridge, even though the virus is larger than the gap width (See the details in Figure S1). To experimentally verify the terahertz response when putting the virus onto the nanogaps, we employed a terahertz time-domain spectroscopy over the frequency range from 0.2 THz to 1.2 THz and observed the resulting redshifts in the resonance peak of the nanogap-loop array coated with the target viruses. To predict the redshifts qualitatively and quantitatively with the coated viruses, we performed full-wave numerical simulation and an analytical calculation based on the modal expansion method [13, 34, 35].

## 2 Results and discussion

**Figure 2:**
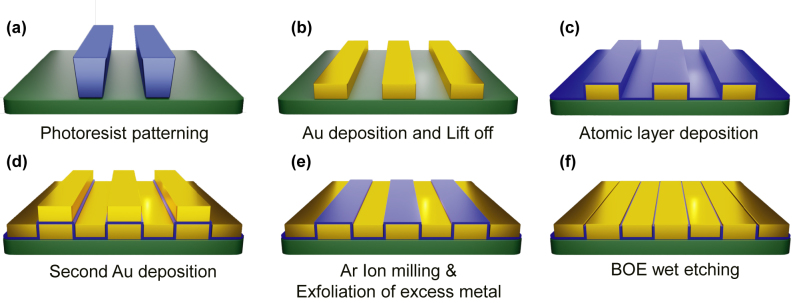
Schematic diagram of the fabrication process of the gold nanogap-loop array. (a) Photoresist patterns are fabricated by conventional photolithography on a silicon wafer. (b) Subsequent Au deposition and lift-off processes are performed to obtain the rectangular shape metal patterns. (c) The patterned metal substrate is conformally coated with a 20 nm thick Al_2_O_3_ layer using atomic layer deposition. (d) The secondary Au deposition is performed to fill inside the rectangular hole arrays. (e) Ar-ion milling is performed before peeling off. The excess metals atop the patterned metal substrate are exfoliated using adhesive tape. (f) Partially remaining Al_2_O_3_ layers are removed by etching with a 6:1 BOE solution for 20 s.

We performed the atomic layer lithography [29, 30] to manufacture a periodic array of gold nanogap-loops with a 20 nm gap width and a 100 μm perimeter. As described in the previous works [29, 36, 37], the detailed fabrication steps are introduced as shown in Figure 2. Conventional photolithography is performed to create patterns on an undoped silicon substrate with a thickness of 500 μm. The n-type photoresist (ma-N-1410) is spin-coated at 3000 RPM for 30 s and subsequently baked at 100 °C for 90 s. After that, the sample is irradiated with ultraviolet light (365 nm wavelength, 10 mW/cm^2^ intensity, 40 s exposure time) using an MA-6 mask aligner and is developed with ma-D 533/S. Cr (5 nm) and Au (95 nm) layers are deposited on the photoresist patterns using an electron-beam evaporator and a lift-off process is performed using an NMP solution. Using an atomic layer deposition, an insulating layer of Al_2_O_3_ with a thickness of 20 nm is conformally covered on the patterned metals, and the thickness of the Al_2_O_3_ layer deposited on the sidewall of the patterns determines the nanogap width. Through secondary Au deposition, the slots are subsequently filled with gold. Finally, Ar-ion milling is performed with an oblique angle of 85° to peel off the excess gold layer more easily with adhesive tape. Further, we performed an additional etching process to remove remained Al_2_O_3_ layer on the top surface of gold using a 6:1 buffered oxide etchant (BOE) solution for 20 s.

**Figure 3:**
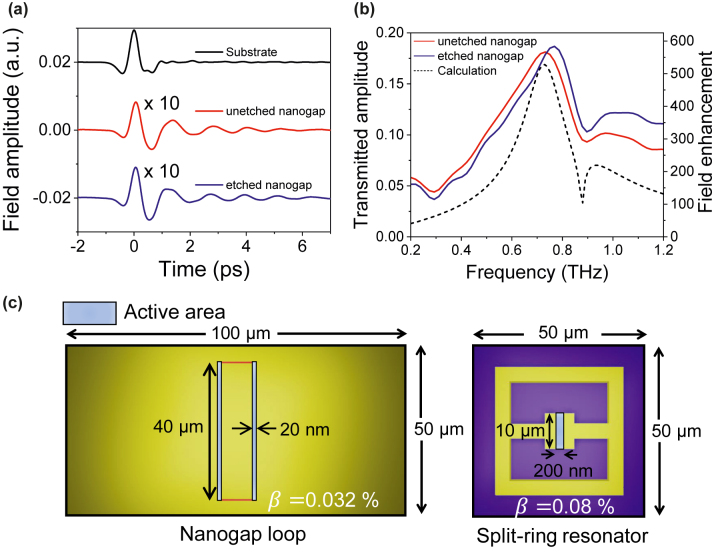
Terahertz time-domain spectroscopy of the gold nanogap-loop array. (a) Time-domain signals passing through a bare silicon substrate (black), the nanogap-loop array before (red), and after (blue) the etching process. (b) Fourier-transformed transmission amplitude and field enhancement spectra of the unetched (red) and the etched (blue) nanogaps. The black dashed curve shows the theoretical calculation using the modal expansion. (c) Schematic diagrams of the coverage ratio (*β*) of the sensing area of the current nanogap-loop**,** and the split-ring resonator which was already reported [7].

To experimentally characterize the gold nanogap-loop arrays, we employed a terahertz time-domain spectroscopy in the frequency range from 0.2 THz to 1.2 THz [38], [39], [40]. We irradiated optical pulses with 800 nm wavelength at GaAs photoconductive antenna for a single-cycle terahertz pulse generation. With a series of parabolic mirrors, terahertz pulse is focused with ∼10 mm^2^ spot area onto nanogap. The transmitted terahertz pulse is guided by two parabolic mirrors and detected by an electro-optic sampling method with a 1 mm-thick (110) oriented ZnTe crystal. Figure 3(a) shows terahertz time traces transmitted through an undoped silicon wafer (black) with a high resistivity (>1000 Ω · cm) as a reference and the 20 nm-wide gap arrays before (red) and after (blue) the BOE etching process. By Fourier-transforming the measured time traces, we converted them to the frequency spectra, which are already normalized by the reference spectrum, as shown in Figure 3(b). It is noted that the resonance frequency of the etched nanogap is slightly blue-shifted because of the reduced thickness of the existing Al_2_O_3_ layer on top of the metal pattern and in the nanogap (Figure 2(f)) [41, 42]. The field enhancement (FE) at the exit of the gap is extracted from the far-field transmitted amplitude using the Kirchhoff integral formalism [43]. It is following the equation, FE = *t*/*β,* where *t* is the far-field transmitted amplitude and *β* is the coverage ratio, resulting in a 580-fold enhancement near the gap at the resonance frequency of 0.77 THz (Figure 3(b)). We also used the modal expansion [13, 34, 44] to theoretically predict the terahertz transmission spectra of the nanogap-loop array. The calculated resonance frequency at 0.73 THz is in good agreement with the experimental results. In addition, the calculation revealed that the dip at 0.88 THz is the result of the interaction between the nanogap resonance and the Rayleigh minima in the periodic array of the nanogap-loop [45, 46]. We note that the spectral broadening in experiments is due to the electric field penetration into the Au film near the nanogap and its resultant absorption; meanwhile the modal expansion method employed the perfect electric conductor without absorption. Furthermore, using the high-density nanogap-loop array [36], the total coverage ratio of 0.032% as an active area (optical hotspot) for sensing the viruses was only 2.5 times smaller than that of the THz split-ring resonators (THz-SRR) in the previous work [7], even though the gap size of 20 nm is 10 times smaller than 200 nm in the THz-SRR structure (Figure 3(c)).

**Figure 4:**
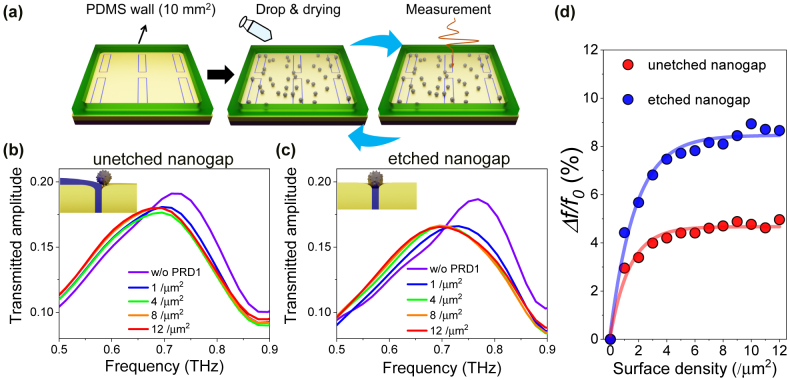
Spectral changes of the virus-coated nanogap-loop array. (a) Schematic diagrams of the drop-castingmethod with the PRD1 viruses. After dropping and drying the PRD1 virus solution inside the PDMS wall, the virus-coated nanogap samples are measured. This cycle (virus coating and THz measurement) is repeated to observe the terahertz spectral changes by increasing the virus density. Terahertz transmitted amplitude spectra for (b) unetched nanogaps and (c) etched nanogaps depending on the selected surface density of the PRD1 virus in the range between 1/μm^2^ and 12/μm^2^. (Insets) Schematic diagrams of the PRD1 virus on the unetched and etched nanogaps. (d) Resonance frequency shift (*Δf/f*_0_) of each sample as a function of the surface density (number of virus particles/μm^2^).

To demonstrate the detection of the virus, the PRD1 virus with an approximate diameter of 60 nm was dispersed on the nanogap-loop array and the terahertz transmission spectrum was measured by varying the surface density of the virus. To uniformly disperse the virus on the surface of nanogap-loop array, PDMS walls with a cross-section area of 10 mm^2^ were built around the nanogap-loop array (Figure 4(a)). The PRD1 virus solution with a volume of 10 μL was dropped onto the nanogap-loop array with PDMS walls and, subsequently, the dropped virus solution was dried at 40 °C for 15 min. The volume density of the virus solution was fixed at 10^9^/mL, and the surface density of the virus was controlled by the number of the repetitive dispersion with the same amount of the solution volume. To figure out the actual number of viruses in the sample, we converted the volume density to the surface density in the same way as in the previous work [7]. With the surface density of 1.0/μm^2^ and the virus solution covering area of 10 mm^2^, we estimate the molar concentration to ∼20 amol for the entire array, corresponding to only ∼10 zmol per one unit cell. Combined with THz near-field imaging spectroscopy with a few micrometers resolution [47], [48], [49], [50], we should be able to observe the resonance peak shift from even smaller quantities of virus particles down to sub-zeptomole. Furthermore, considering only the actual sensing area, the average number of viruses participating in sensing is estimated to be below 100 (For the details, see the following full-wave numerical simulation results).

Figure 4(b) and (c) show the terahertz transmission amplitude spectra for the unetched and etched nanogaps as increasing the surface density of the PRD1 virus from 1/μm^2^ to 12/μm^2^. We clearly observed redshifts in the resonance peaks of the 20 nm-gap loop array even with the virus density of 1/μm^2^. The resonance peak shifts are because the virus particles, which are tightly fitted into the nanogap, strongly perturbed the resonant mode of the rectangular nanogap-loops [32, 51]. In addition, we verified that the resonance frequency of the transmission amplitude spectrum returned to its original frequency by applying a fungicide treatment [14] with sodium hypochlorite solution which easily removes the PRD1 virus particle from the surface (See the details in Figure S2).

Figure 4(d) shows 3–4% redshifts after coating a virus solution with the surface density of 1/μm^2^, and the resonance frequency shifts (Δ*f/f*_0_) gradually saturate to maximum values of 4.9% and 8.9% for the unetched and etched nanogaps, respectively. This difference originates from the rapidly decaying evanescent field on the surface of the nanogap region, resulting in less sensitivity to the change of the local index of refraction in the unetched nanogap with the 20 nm thick Al_2_O_3_ layer [32, 52]. Compared to the THz-SRR sensing with a gap width of 200 nm [7], the virus sensing capability of the etched 20 nm nanogap-loop array has been enhanced to 160% of the maximum frequency shift and 400% of the frequency shift per single virus particle. These results indicate that tightly attaching the virus particle to the nanogap region forming the bridge between the two metal surfaces shows better sensitivity compared to the one with the wider gap with the loose-fitting particles [7, 8, 51]. It is worth noting that our new nanogap platform provides a two-dimensional-like optical hotspot, facilitating the detection of ultrasmall quantity of viruses (Figure 4(c)). The opening of the two-dimensional-like (2D-like) optical hotspot allows us to access the resonance mode by effectively deforming the electric field on the surface of the nanogap, resulting in the shift of the resonance frequency. Furthermore, using advanced optical or electrical tweezing techniques [53], [54], [55], it is possible to place viruses in the desired nanogap region and enhance the virus detection sensitivity in sub-zeptomole range. In the current drop-drying method, the virus particles were randomly distributed on the nanogap-loop array including the weak electric field area. However, the tweezing techniques may allow us to place the virus in the nanogap region with strong electric fields which facilitates sub-zeptomolar detection.

**Figure 5:**
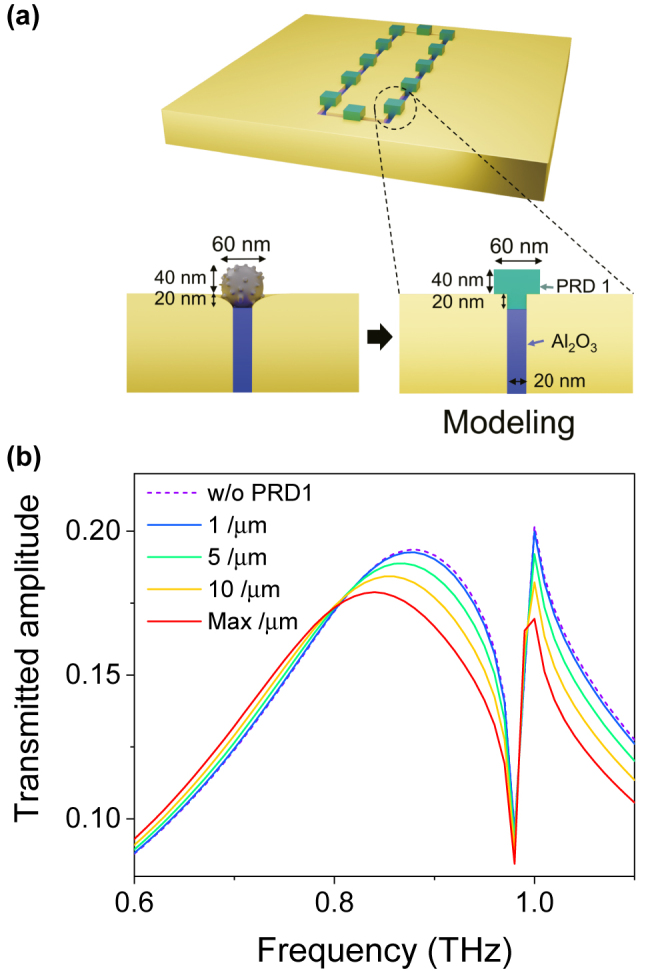
Full-wave numerical simulations. (a) Schematic illustrations of the simulation modeling. The spherical shape of the PRD1 virus has been changed to a T-shape in order to minimize the computing time of the simulation. (b) The transmission amplitude spectra of the 20 nm gap depending on their PRD1 virus densities. Here the density of the virus particle is defined by the number of viruses per unit length in the nanogap-loop.

To elucidate the sensitivity enhancement in the nanogap-loop with ultrasmall amount of the virus particles, we performed the full-wave numerical simulations by using the Finite-element method (See the details for optical simulations in the method). The tightly attached virus particles in the etched nanogap region contact with three faces; the top surface of Al_2_O_3_ gap-filling material and the two slanted gap surfaces of the metal (Figure 4(c)). To consider such a structure, we modeled the spherical shape virus to a hexahedron shape with a bump fit to the nanogap (Figure 5(a)). The refractive index of the virus particle was set to 1.87 + 0.13i from the earlier result [7]. Figure 5(b) shows the calculated transmission amplitude spectrum with varying the density of the virus particles and the resultant redshifts of the resonance frequency with the increase of the virus density. With the fully covered case with the maximum density, the resonance frequency shift is saturated as shown in the experiments. It is emphasized that although the virus particles were uniformly drop-cast over the whole area, the actual amount of virus particles participating might only be a few hundreds near the nanogap-loop.

We verified this noticeable result analytically by placing a layer with a refractive index of 1.87 + 0.13i, as same as that of viruses, over the nanogap (See the details in Supporting Information). By the virus layer, the analytically calculated resonance frequency shift was 30 GHz and transmission amplitude decreased by 12.5% (Figure S3). Although the partial refractive index change inside the nanogap (Δ*n*_*2*_) was not considered in the analytic calculation, we emphasize that there is not a big difference between the numerical simulations with the virus particles and the analytic calculations with the virus layer, indicating that the local index changes near the 2D-like optical hotspot mostly determines the change of the resonant transmission spectrum.

## 3 Conclusions

We fabricated virus-sized gold nanogaps with a gap width of 20 nm using the atomic layer lithography and implemented an ultra-sensitive virus detection platform working in the THz frequency region. The large-area nanogap-loop array allowed a 580-fold field enhancement, enabling extreme light–matter interactions near the gap. Through the etching process, it was possible to remove the existing Al_2_O_3_ layer on top of the first metal pattern and part of the inner layer between the two metals, wherein the virus particle can be tightly attached to the nanogap as bridging the gap between the two metals [56]. We consequently observed a factor of two increase in the redshift of the resonant peak compared to the unetched gap sample, leading to the giant sensitivity enhancement of 400% of the resonance frequency shift per single virus particle compared to the previous work [7]. In full-3D simulations and modal expansions, the results were in good agreement with the results of the experiments and showed that the refractive index change near the nanogap, forming an optical hotspot area in two dimensions, largely determined the resonant transmission spectrum. Further sensitivity could be improved by matching the nanogap-loops to one of the viruses’ absorption frequencies, associated with their intermolecular vibrational modes, and fingerprinting of the target virus [13, 55, 57, 58]. With our high-sensitivity, rapid-detection virus sensing technique, we offer a new method of mass-screening for deadly and contagious viruses, including COVID-19.

## 4 Methods

**Optical simulations**. The full-wave optical simulations were performed to analyze an ultrasmall quantity virus detection in the nanogap-loop array by using the finite-element method (COMSOL Multiphysics, RF module). The nanogap-loop array was formed in the 100 nm thick gold film on a SiO_2_-coated silicon substrate. The lengths of the loop nanogap in the *x*- and *y*-direction were 10 and 40 μm, respectively. The gap width was 20 nm. The pitches of the array were 50 and 100 μm in the *x*- and *y*-direction, respectively. The gap region with a height of 80 nm from the substrate was filled with Al_2_O_3_, and the rest of the gap region was filled with air for modeling the etched and wider gap region. The refractive indices of Al_2_O_3_, SiO_2,_ and Si in the terahertz region were 2.35, 2.1, and 3.4, respectively, and the dielectric constant of gold was estimated by the Drude model, *ε*(*ω*) = 1 – *ω*_p_^2^/(*ω* ^2^+i*ωγ*_p_) with *ω*_p_ of 2730 THz and *γ*_p_ of 19.4 THz. The *x*-polarized plane wave was impinging from the substrate, and the transmitted amplitude was measured from the air.

To examine the virus detection, the hexahedron shape virus particles with a bump fit the nanogap were placed on top of the gap region. The width, height, and depth of the virus particles were 60, 60, and 40 nm, respectively (Figure 5(a)). In addition, the air-filled gap region was filled with the bump in order to model the sphere-shaped virus particle embedded into the slanted gap region. The width, height, and depth of the bump were 20, 60, and 20 nm, respectively. To analyze the resonance peak shift with varying the density of the virus particle, the virus particles were periodically placed along the loop of the nanogap, with the inter-distance of 0, 100, 200, and 1000 nm. The refractive index of virus material was 1.87 + 0.13i [7].

The different length scales in the gap width (20 nm), the length of nanoresonators (40 μm), and the wavelength (600 μm at 0.5 THz) demand huge amounts of computational resources. To resolve it, we applied a multi-scale meshing method based on the physical origin of the resonance.

## Supplementary Material

Supplementary Material Details

## References

[1] Smyrlaki I., Ekman M., Lentini A. (2020). Massive and rapid COVID-19 testing is feasible by extraction-free SARS-CoV-2 RT-PCR. *Nat. Commun.*.

[2] Mackay I. M., Arden K. E., Nitsche A. (2002). Real-time PCR in virology. *Nucleic Acids Res*..

[3] Guglielmi G. (2020). Fast coronavirus tests are coming. *Nature*.

[4] Saleh O. A., Sohn L. L. (2003). Direct detection of antibody-antigen binding using an on-chip artificial pore. *Proc. Natl. Acad. Sci. U.S.A.*.

[5] Larremore D. B., Wilder B., Lester E. (2021). Test sensitivity is secondary to frequency and turnaround time for COVID-19 screening. *Sci. Adv.*.

[6] Murphy C. J., Chang H. H., Lotsch P. F. (2019). Virus-sized gold nanorods: plasmonic particles for biology. *Acc. Chem. Res.*.

[7] Park S. J., Cha S. H., Shin G. A., Ahn Y. H. (2017). Sensing viruses using terahertz nano-gap metamaterials. *Biomed. Opt. Express*.

[8] Lee D. K., Kang J. H., Kwon J. (2017). Nano metamaterials for ultrasensitive Terahertz biosensing. *Sci. Rep.*.

[9] Neutze R., Wouts R., Spoel D., Weckert E., Hajdu J. (2000). Potential for biomolecular imaging with femtosecond X-ray pulses. *Nature*.

[10] Lee H. J., Lee J. H., Moon H. S. (2012). A planar split-ring resonator-based microwave biosensor for label-free detection of biomolecules. *Sens. Actuators B Chem.*.

[11] Zhang S., Wong C. L., Zeng S. (2021). Metasurfaces for biomedical applications: imaging and sensing from a nanophotonics perspective. *Nanophotonics*.

[12] Zanchetta G., Lanfranco R., Giavazzi F., Bellini T., Buscaglia M. (2017). Emerging applications of label-free optical biosensors. *Nanophotonics*.

[13] Park H. R., Ahn K. J., Han S., Bahk Y. M., Park N., Kim D. S. (2013). Colossal absorption of molecules inside single terahertz nanoantennas. *Nano Lett*..

[14] Park S. J., Hong J. T., Choi S. J. (2014). Detection of microorganisms using terahertz metamaterials. *Sci. Rep.*.

[15] Seo M., Park H. R. (2020). Terahertz biochemical molecule-specific sensors. *Adv. Opt. Mater.*.

[16] Jun S. W., Ahn Y. H. (2022). Terahertz thermal curve analysis for label-free identification of pathogens. *Nat. Commun.*.

[17] Yoon S. A., Cha S. H., Jun S. W. (2020). Identifying different types of microorganisms with terahertz spectroscopy. *Biomed. Opt. Express*.

[18] Olsen R. H., Siak J. S., Gray R. H. (1974). Characteristics of Prd1, a plasmid-dependent broad host range DNA bacteriophage. *J. Virol.*.

[19] Woo M. H., Hsu Y. M., Wu C. Y., Heimbuch B., Wander J. (2010). Method for contamination of filtering facepiece respirators by deposition of MS2 viral aerosols. *J. Aerosol Sci.*.

[20] Bar-On Y. M., Flamholz A., Phillips R., Milo R. (2020). SARS-CoV-2 (COVID-19) by the numbers. *eLife*.

[21] Beruete M., Jauregui-Lopez I. (2020). Terahertz sensing based on metasurfaces. *Adv. Opt. Mater.*.

[22] Shen S., Liu X., Shen Y. (2022). Recent advances in the development of materials for terahertz metamaterial sensing. *Adv. Opt. Mater.*.

[23] Wang Y., Han Z., Du Y., Qin J. (2021). Ultrasensitive terahertz sensing with high-Q toroidal dipole resonance governed by bound states in the continuum in all-dielectric metasurface. *Nanophotonics*.

[24] Zhang C., Xue T., Zhang J. (2022). Terahertz toroidal metasurface biosensor for sensitive distinction of lung cancer cells. *Nanophotonics*.

[25] Sengupta R., Khand H., Sarusi G. (2022). Terahertz impedance spectroscopy of biological nanoparticles by a resonant metamaterial chip for breathalyzer-based COVID-19 prompt tests. *ACS Appl. Nano Mater.*.

[26] Lee S. H., Lee Y. K., Lee S. H., Kwak J., Song H. S., Seo M. (2022). Detection and discrimination of SARS-CoV-2 spike protein-derived peptides using THz metamaterials. *Biosens. Bioelectron.*.

[27] Kang J. H., Kim D. S., Seo M. (2018). Terahertz wave interaction with metallic nanostructures. *Nanophotonics*.

[28] Amin M., Siddiqui O., Abutarboush H., Farhat M., Ramzan R. (2021). A THz graphene metasurface for polarization selective virus sensing. *Carbon*.

[29] Chen X., Park H. R., Pelton M. (2013). Atomic layer lithography of wafer-scale nanogap arrays for extreme confinement of electromagnetic waves. *Nat. Commun.*.

[30] Bahk Y. M., Kim D. S., Park H. R. (2019). Large-area metal gaps and their optical applications. *Adv. Opt. Mater.*.

[31] Jeong J., Kim D., Park H. R. (2018). Anomalous extinction in index-matched terahertz nanogaps. *Nanophotonics*.

[32] Park H. R., Chen X., Nguyen N. C., Peraire J., Oh S. H. (2015). Nanogap-enhanced terahertz sensing of 1 nm thick (λ/10^6^) dielectric films. *ACS Photonics*.

[33] Abrescia N. G. A., Cockburn J. J. B., Grimes J. M. (2004). Insights into assembly from structural analysis of bacteriophage PRD1. *Nature*.

[34] Garcia-Vidal F. J., Moreno E., Porto J. A., Martin-Moreno L. (2005). Transmission of light through a single rectangular hole. *Phys. Rev. Lett.*.

[35] Kang J. H., Choe J. H., Kim D. S., Park Q. H. (2009). Substrate effect on aperture resonances in a thin metal film. *Opt. Express*.

[36] Park H. R., Namgung S., Chen X., Oh S. H. (2015). High-density metallic nanogap arrays for the sensitive detection of single-walled carbon nanotube thin films. *Faraday Discuss*..

[37] Park H. R., Namgung S., Chen X. (2015). Perfect extinction of terahertz waves in monolayer graphene over 2-nm-Wide metallic apertures. *Adv. Opt. Mater.*.

[38] Nahata A., Weling A. S., Heinz T. F. (1996). A wideband coherent terahertz spectroscopy system using optical rectification and electro-optic sampling. *Appl. Phys. Lett.*.

[39] Vanexter M., Fattinger C., Grischkowsky D. (1989). Terahertz time-domain spectroscopy of water-vapor. *Opt. Lett.*.

[40] Wu Q., Litz M., Zhang X. C. (1996). Broadband detection capability of ZnTe electro-optic field detectors. *Appl. Phys. Lett.*.

[41] Jeong J., Yun H. S., Kim D. (2018). High contrast detection of water-filled terahertz nanotrenches. *Adv. Opt. Mater.*.

[42] Kim D., Jeong J., Choi G. (2018). Giant field enhancements in ultrathin nanoslots above 1 terahertz. *ACS Photonics*.

[43] Kyoung J. S., Seo M. A., Park H. R., Ahn K. J., Kim D. S. (2010). Far field detection of terahertz near field enhancement of sub-wavelength slits using Kirchhoff integral formalism. *Opt. Commun.*.

[44] Garcia-Vidal F. J., Martin-Moreno L., Moreno E., Kumar L. K. S., Gordon R. (2006). Transmission of light through a single rectangular hole in a real metal. *Phys. Rev. B*.

[45] Park H. R., Park Y. M., Kim H. S. (2010). Terahertz nanoresonators: giant field enhancement and ultrabroadband performance. *Appl. Phys. Lett.*.

[46] Lee J. W., Seo M. A., Kim D. S. (2006). Fabry-Perot effects in THz time-domain spectroscopy of plasmonic band-gap structures. *Appl. Phys. Lett.*.

[47] Blanchard F., Doi A., Tanaka T. (2011). Real-time terahertz near-field microscope. *Opt. Express*.

[48] Heo C., Ha T., You C. (2020). Identifying fibrillization state of A beta protein via near-field THz conductance measurement. *ACS Nano*.

[49] Wade C. G., Šibalić N., de Melo N. R., Kondo J. M., Adams C. S., Weatherill K. J. (2017). Real-time near-field terahertz imaging with atomic optical fluorescence. *Nat. Photonics*.

[50] Ishihara K., Ohashi K. (2006). Terahertz-wave near-field imaging with subwavelength resolution using surface-wave-assisted bow-tie aperture. *Appl. Phys. Lett.*.

[51] Bahk Y. M., Kim K. H., Ji G., Ahn K. J., Kim D. S., Park H. R. (2022). Detection of single nanoparticles inside a SingleTerahertz resonator. *Adv. Photonics Res.*.

[52] Park H. R., Koo S. M., Suwal O. K. (2010). Resonance behavior of single ultrathin slot antennas on finite dielectric substrates in terahertz regime. *Appl. Phys. Lett.*.

[53] Yoo D., Barik A., de León-Pérez F. (2021). Plasmonic split-trench resonator for trapping and sensing. *ACS Nano*.

[54] Lee G., Yu E. S., Ryu Y. S., Seo M. (2022). The perspectives of broadband metasurfaces and photo-electric tweezer applications. *Nanophotonics*.

[55] Burkhartsmeyer J., Wang Y., Wong K. S., Gordon R. (2020). Optical trapping, sizing, and probing acoustic modes of a small virus. *Appl. Sci.*.

[56] Park H. R., Bahk Y. M., Choe J. H. (2011). Terahertz pinch harmonics enabled by single nano rods. *Opt. Express*.

[57] Tsen K. T., Dykeman E. C., Sankey O. F., Lin N. T., Tsen S. W. D., Kiang J. G. (2006). Observation of the low frequency vibrational modes of bacteriophage M13 in water by Raman spectroscopy. *Virol. J.*.

[58] Jeong J., Kim D. S., Park H. R. (2022). Beyond-hot-spot absorption enhancement on top of terahertz nanotrenches. *Nanophotonics*.

