# Supplementary material for: Terahertz virus-sized gold nanogap sensor
Source: Nanophotonics. 2023 Jan 3;12(1):147–54. doi: 10.1515/nanoph-2022-0706 (PMC11501242; doi:10.1515/nanoph-2022-0706)
Supplement: Supplementary file 1 — Supplementary Material Details [file j_nanoph-2022-0706_suppl.pdf]

Supporting Information

Terahertz virus-sized gold nanogap sensor

Gangseon Ji[1], Hwan Sik Kim[2], Seong Ho Cha[2], Hyoung-Taek Lee[1], Hye Ju Kim[2], Sang Woon Lee[2], Kwang Jun Ahn[2], Kyoung-Ho Kim[3,‡], Yeong Hwan Ahn[2,†], and Hyeong-Ryeol Park[1,*]

[1]Department of Physics, Ulsan National Institute of Science and Technology (UNIST), Ulsan 44949, Republic of Korea

[2]Department of Physics and Department of Energy Systems Research, Ajou University, Suwon 16499, Republic of Korea

[3]Department of Physics and Research Institute for Nanoscale Science and Technology, Chungbuk National University, Cheongju 28644, Republic of Korea

E-mails: [‡]kyoungho@chungbuk.ac.kr, [†]ahny@ajou.ac.kr, [*]nano@unist.ac.kr

1. Field emission scanning electron microscopy (FE-SEM) images of virus-coated nanogap-loop array

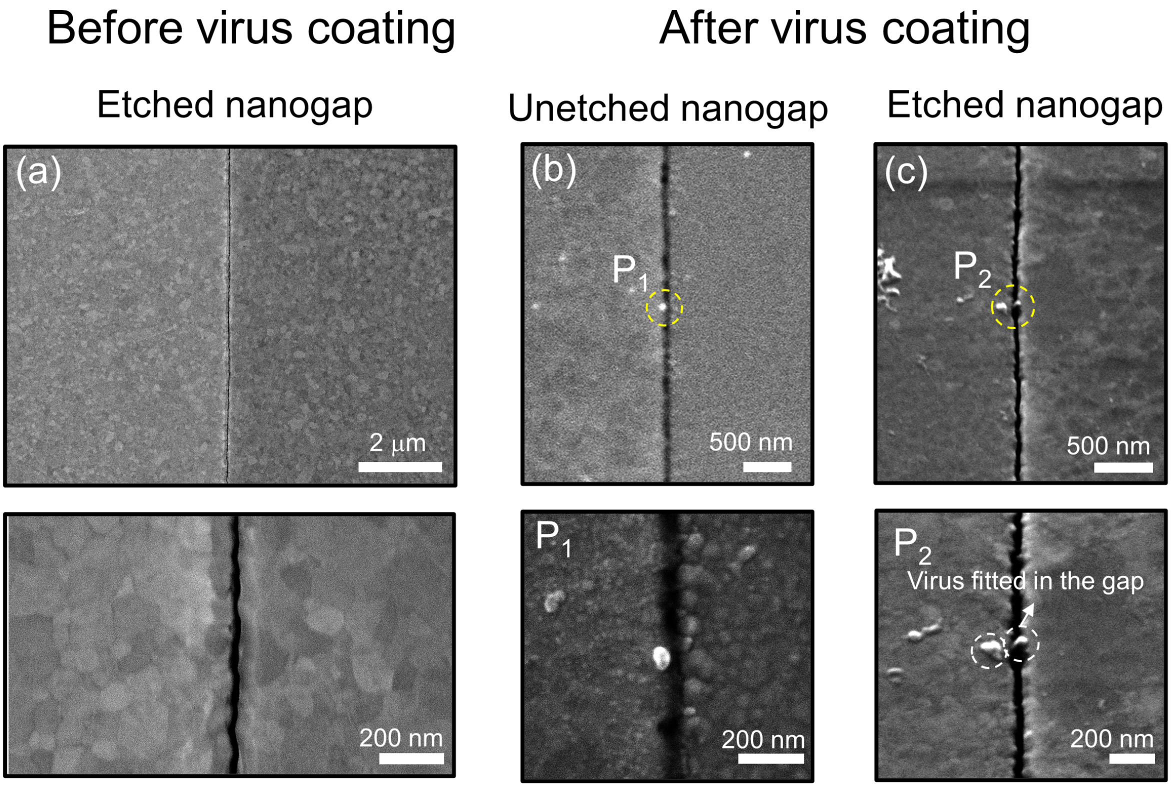

Figure S1. FE-SEM images of one side of the nanogap-loop with the 20 nm gap width (a) before virus coating for the etched gap, (b, c) after virus coating for the unetched and etched gap. The 120 μl of virus solution was coated on the 10 mm^2 surface area, corresponding estimated surface density is $12/\mu m^2$. We performed a Pt coating on the entire sample to avoid the electric charging effect from the dielectric virus particle. For that reason, the virus may appear slightly larger.

2. Normalized transmitted amplitude spectra of the nanogap sample before and after cleaning with fungicide treatment

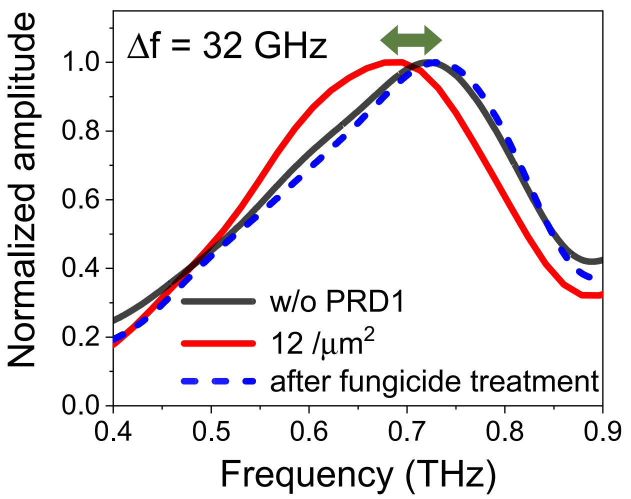

Figure S2. Normalized transmitted amplitude spectra of the unetched nanogap sample only (black solid), with PRD1 viruses of 12/μm^2 (red solid), and after fungicide treatment (blue dash). We clearly observed that the resonance frequency of virus-coated nanogap returned to its original resonance frequency of 0.72 THz after fungicide treatment.

3. Modal expansion method

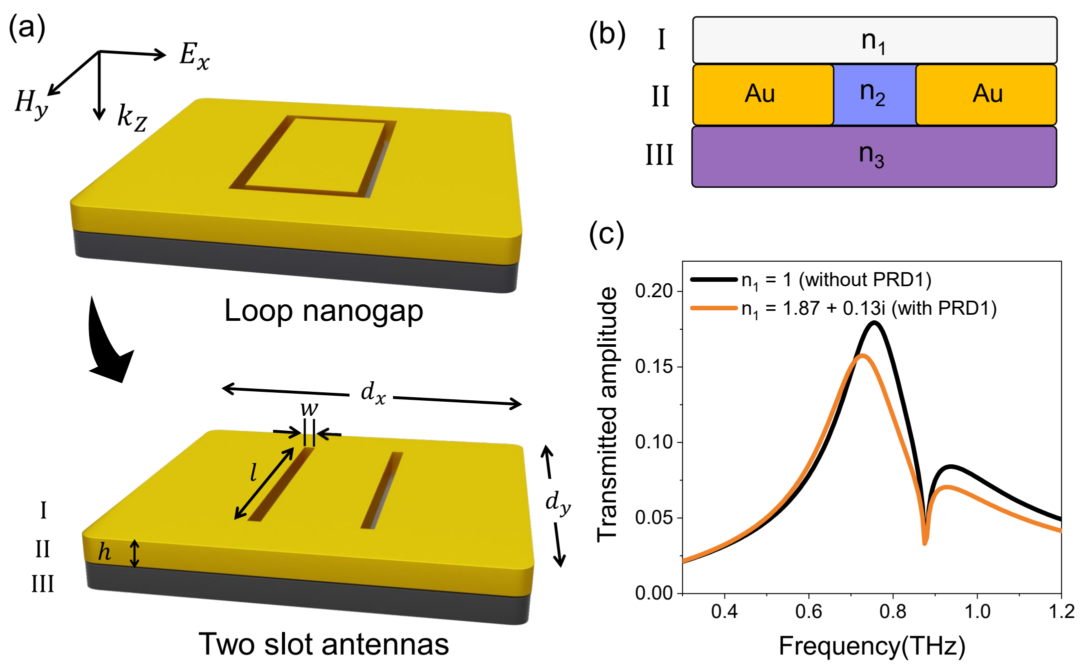

Figure S3. (a) The geometry of nanogap slot antenna applied to the modal expansion calculations. (b) Simplified schematic of a cross-sectional view of the nanogap slot antenna. Here, n_1 is the refractive index of air or the effective complex refractive indices of PRD1 suggested in the previous work [1]. n_2 and n_3 are the refractive indices of the Al_2O_3 layer between the gold structures and the undoped silicon substrate, respectively. (c) The transmitted amplitude spectra of a 20 nm-wide gap slot antenna with and without the PRD1 layer are obtained using the analytical model based on the modal expansion.

The optical properties of the nanogap structure are theoretically obtained by applying the modal expansion method combined with a gap plasmon effect [2, 3]. Surface plasmon at a metal-dielectric interface cannot be observed at low frequencies (including terahertz frequency), since there is an indistinguishable dispersion deviation between surface plasmon and light line. In contrast, if the gap size is similar to or smaller than the skin depth of gold, two surface plasmons can be coupled in a metal-insulator-metal structure. Then coupled surface plasmon (i.e., gap plasmon) starts to dominate with a dispersion relation as follows [4]:

$$tanh\frac{k_d w}{2} = -\frac{k_m \epsilon_d}{k_d \epsilon_m}, \qquad {k_i}^2 = \beta^2 - {k_0}^2 \epsilon_i \ (\mathrm{i} = \mathrm{m}, \mathrm{d})$$

where k_0 is the wavevector of light in the vacuum, k_i is the wavevector component perpendicular to the metal-dielectric surface, w is the width of the dielectric (the Al_2O_3 region in Figure S2(b)), β is the propagation constant, ε_i is the permittivity of the material i, and the subscripts d and m denote dielectric and metal.

Our loop nanogap can be assumed as the two slot antennas with the same gap width of w. As shown in Figure S2 (a), we defined the region I, II, and III as follow: (I) above the slot antenna, (II) inside the slot antenna, and (III) below the slot antenna. We can derive the electric field amplitude at the exit of gap by solving the boundary conditions at each interface,

$$E_{gap} = \frac{I_0 G_V}{(G_I - \Sigma)(G_{III} - \Sigma) - G_V{}^2}$$

where G_I and G_{III} are the coupling constants of the gap mode, and the constants Σ and G_V depend on the propagation constant of the gap. Outside the gap, the electric and magnetic fields are expressed as

$$E_x(\vec{r}) = E_{gap} \times \sum_{k_x,k_y} \frac{J(k_x,k_y)}{dxdy} exp(i\vec{k}\cdot\vec{r})$$

$$H_y(\vec{r}) = \frac{E_{gap}}{Z_0} \times \sum_{k_x,k_y} \frac{J(k_x,k_y)}{dxdy}\frac{k_x^2+k_z^2}{k_0 k_z} exp(i\vec{k}\cdot\vec{r})$$

$$J(k_x,k_y) = sinc(k_x w/2)\left\{sinc\left(\frac{\pi+k_y l}{2}\right) + sinc\left(\frac{\pi-k_y l}{2}\right)\right\}$$

Here, k_x and k_y are $2\pi/dx$ and $2\pi/dy$ multiplied by an integer, respectively. And Z_0 is the impedance of free space. To observe the spectral changes after adding the top virus layer, the refractive index of region I is modified to the effective value of $n_I = 1.87 + 0.13i$ [1]. Although the thickness of the virus is set to a half-infinite layer that greatly exceeds the gap size of 20 nm, the shift of resonance peak saturates to a maximum value of about 30 GHz in Figure S2(c).

References

[1] S. J. Park et al., "Sensing viruses using terahertz nano-gap metamaterials," *Biomed. Opt. Express,* vol. 8, p. 3551, 2017.

[2] F. J. Garcia-Vidal et al., "Transmission of light through a single rectangular hole," *Phys. Rev. Lett.,* vol. 95, p. 103901, 2005.

[3] F. J. Garcia-Vidal et al., "Transmission of light through a single rectangular hole in a real metal," *Phys. Rev. B,* vol. 74, p. 153411, 2006.

[4] S. A. Maier, Plasmonics: fundamentals and applications, Springer Science & Business Media, 2007.
